# Efficacy assessment of acupuncture in improving symptoms of uterine fibroids: A randomized controlled trial

**DOI:** 10.1097/MD.0000000000020016

**Published:** 2020-05-01

**Authors:** Min-Qin Zheng, Cai Weng, Wei Hu, Chong-Qing Shen, Yun Tao, Zheng-Wei Pan

**Affiliations:** a Fuzhou Hospital of Traditional Chinese Medicine, Fuzhou 350013; b Fujian University of Traditional Chinese Medicine, Fuzhou 350100; c The Second Affiliated People's Hospital of Fujian University of Traditional Chinese Medicine, Fuzhou 350013; d Department of Dermatology, Huabei Petroleum General Hospital, Renqiu 062550; e Fujian University of Traditional Chinese Medicine Subsidiary Rehabilitation Hospital, Fuzhou 350013, China.

**Keywords:** acupuncture, protocol, randomized controlled trial, uterine fibroids

## Abstract

**Introduction:**

Uterine fibroids are a common benign genital tumor disease in gynecological diseases. It is mainly a change in physical function caused by the growth of smooth muscle cells in the factor uterus. Modern medicine's treatment of this disease is based on the dependence of uterine fibroids on sex hormones. Treatment with antiprogestin and estrogen drugs can reduce the volume of fibroids or slow the rate of increase in volume, thereby achieving the goal of alleviating clinical symptoms. In order to meet the needs of the majority of women of childbearing age and to maintain fertility, acupuncture treatment of uterine fibroids has a broad prospect for development.

**Methods/design:**

This study plans to select 60 cases that meet the corresponding selection criteria. According to the random principle, they will be divided into intervention group and control group, with 30 cases in each group. The general information, fibroid size, and TCM syndrome scores of the two groups of patients will be compared before treatment. In terms of treatment, the intervention group will be given acupuncture combined therapy; the control group will be given Chinese patent medicine. The treatment cycles in both groups will be three menstrual cycles. After the treatment is completed, the data of the relevant curative effect indicators are analyzed by using SPSS software to draw conclusions.

**Discussion:**

We aim to provide higher evidence-based medical evidence for acupuncture treatment of uterine fibroids.

**Trial registration:**

ClinicalTrials.gov, ChiCTR2000030438, Registered on March 01, 2020.

## Abstract 2

Supplemental Digital Content is available in the text

## 1 Introduction

Uterine fibroids (UF) are a common benign genital tumor disease in gynecological diseases. It is mainly a change in physical function caused by the growth of smooth muscle cells in the factor uterus.^[1]^ Common clinical symptoms include abdominal pain, irregular menstruation, and increased vaginal discharge. In severe cases, anemia may occur and even secondary infertility. Some patients also appear asymptomatic.^[2]^ Modern medicine has not stopped exploring the pathogenesis of. So far, as modern medical research confirms, the pathogenesis of UF has obvious ethnic differences and genetic susceptibility.^[3]^ A large number of studies have shown that UF are relatively high in women of childbearing age, but rare in postmenopausal women and pre-maturity. UF generally shrink to some extent after menopause or even disappear, but fibroids can grow properly when a woman is pregnant. In summary, the pathogenesis of UF is not completely clear. Most experts hold that the occurrence of UF may be affected by heredity, and it is related to the regulation of growth factors.^[4,5]^ Among the various schemes for treating UF at this stage, surgical treatment is the main treatment method. Transabdominal hysterectomy is the most traditional, classic, and relatively mature technique. However, this method brings huge surgical trauma. Therefore, it is only suitable for patients with no fertility requirements or suspected malignancy.^[6]^ Modern medical treatment methods for this disease mainly include surgical treatment, drug treatment, radiofrequency ablation, and so on. Although treatments vary, they are not suitable for all patients. And each has many side effects and limitations.

Patients’ needs for quality of life and fertility are getting higher and higher, and it is urgent to explore the pathogenesis and how to effectively treat UF.^[7,8]^ Traditional Chinese and Western medicine currently have a variety of treatment options for UF. Western medicine is mainly divided into surgical treatment and drug treatment. Although surgical treatment is fast and effective, it has a large trauma to the uterus, which is not conducive to the fertility needs of patients, and drug treatment is very easy to form dependence.^[9]^ Traditional Chinese medicine (TCM) treatment is more diverse, and acupuncture, Chinese herbal medicine, etc have a certain effect on the disease. However, the lack of a large sample of clinical evaluation and uniform evaluation standards has greatly limited the promotion of TCM. In recent years, the curative effect of acupuncture treatment on UF has attracted the attention of medical workers, and related clinical research has been increasing.^[10]^ However, the quality of clinical protocol design and literature reports still needs to be improved, and the evidence of efficacy is insufficient. More high-quality studies are needed to evaluate the efficacy and safety of acupuncture in improving the symptoms of patients with UF. This trial intends to adopt a randomized, patient-blind, and blinded efficacy evaluator and data processor design scheme to compare the difference in the efficacy of acupuncture and fake needles to improve the symptoms of UF, and provide new evidence for acupuncture treatment of UF.

## 2 Methods/design

### 2.1 Study design and settings

**Figure 1:**
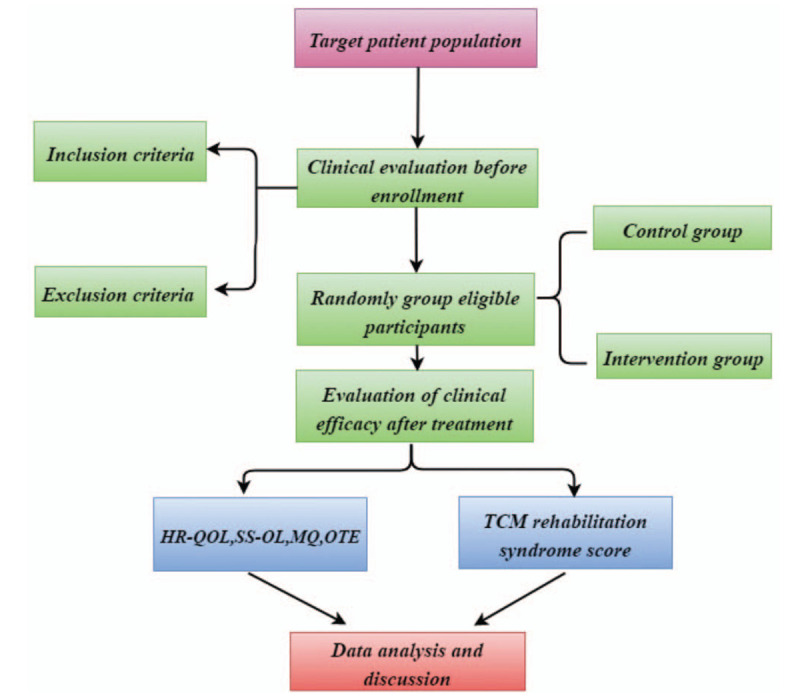
Study design flow chart.

A brief flowchart of the entire study is shown in Figure 1. We will perform a two-group, randomized, single-blind, placebo-controlled, multi-center trial that will evaluate the efficacy and safety of acupuncture for patients with UF. This study will use a completely random grouping and parallel control observation design method. We will ensure the balance of the baseline data of the two groups through a sufficient sample size and a completely randomized grouping method. The protocol includes elements recommended in the Standard Protocol Items: Recommendations for Interventional Trials checklist (Supplemental Digital Content, Appendix 1).

### 2.2 Participants

**Figure 2:**
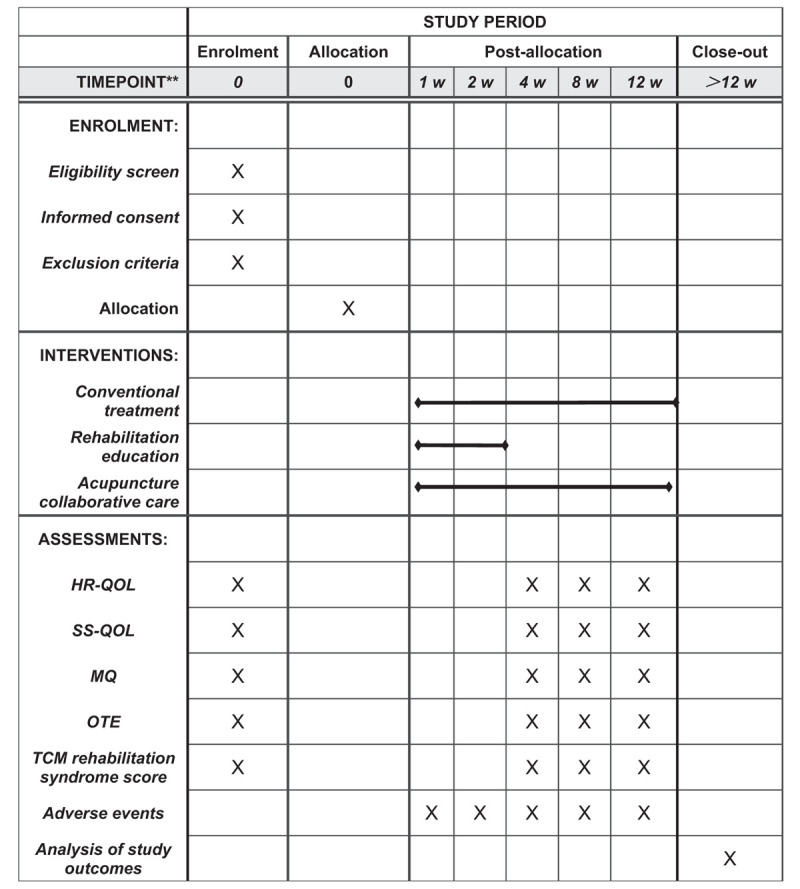
SPIRIT figure for the schedule of enrollment, interventions, and assessments. HR-QOL = The health-related quality of life of uterine fibroid, MQ = Menorrhagia Questionnaire, OTE = Overall Recovery Self-Scoring, SPIRIT = Standard Protocol Items: Recommendations for Interventional Trials, SS-QOL = symptom severity of quality uterine fibroid, TCM = Traditional Chinese medicine.

All cases in this study will come from Gynecology clinics and wards. This study intends to include a total of 60 samples. B-ultrasounds suggest that uterine fibroids have a maximum diameter of <4 cm and meet the following study standards. Eligible cases will be randomly divided into treatment group and intervention group according to the digital randomization grouping method (Fig. 2).

#### 2.2.1 Diagnostic criteria

Refer to the Canadian Society of Obstetricians and Gynecologists (SOGC) clinical practice guidelines and the 2009 Hong Kong Academy of Obstetrics and Gynecology (HKCOG) guidelines.

(1) Increased menstrual flow and prolonged periods, lower abdominal masses;
(2) Pelvic pain and other common lower abdominal bloating;
(3) Back pain and menstrual symptoms;
(4) Pelvic compression symptoms, intestinal dysfunction and bladder symptoms, such as frequent urination and urgency;
(5) Meet the pelvic B ultrasound diagnosis.

The diagnostic criteria of TCM will refer to the dialectical criteria for uterine fibroids in the “*Traditional Chinese Medicine Gynecology*” published by China TCM Press, 3rd edition, 2012. Main symptoms of TCM: lower abdominal mass; Secondary symptoms: lower abdomen pain, refusing to press, dry mouth, do not want to drink, delayed or dripping menstruation, more or less menstrual flow, dark purple, thick and lumpy skin Shaoze or dull expression.

#### 2.2.2 Inclusion criteria

(1) Female patients aged 18 to 55 (middle and young);
(2) Meet the western medical diagnostic standards for UF;
(3) The uterine body does not exceed the size of pregnancy at 10 weeks, and the diameter of the tumor is <4 cm;
(4) Did not use other drugs that have an impact on the results of this study within 1 month before enrollment;
(5) Sign the informed consent.

#### 2.2.3 Exclusion criteria

Patients will be excluded if they meet the following criteria:

(1) Patients with ovarian tumors, adenomyosis, uterine malignant tumors, and urinary tract infections;
(2) Women during pregnancy or lactation;
(3) Patients who have taken hormonal drugs in the past 3 months, or have been treated with this research protocol;
(4) People with severe heart, liver, and kidney dysfunction and mental disorders;
(5) Subjects participating in other clinical studies;

#### 2.2.4 Case rejection or shedding criteria

After inclusion, if we find that it does not meet the inclusion criteria or does not operate according to this plan, we will reject it. Before the statistics are collected, the statistician and the main investigator should discuss whether to eliminate the case.

#### 2.2.5 Suspension criteria

Patients who have other illnesses during the case study, or who cannot continue acupuncture treatment due to halo acupuncture, and who withdraw on their own should be regarded as falling off.

### 2.3 Interventions

#### 2.3.1 Control group

We will give Chinese medicine treatment. Women's Yangxue Pills (Gansu Hexi Pharmaceutical Co., Ltd., 0.34 g per 10 pills, 4 g per bag, approval number: Sinopharm Standard Z20103019). How to take: Take it orally two times a day, one sachet each time, and take 20 minutes after a meal. A menstrual cycle is a course of treatment, and even takes three courses. Precautions:

(1) The diet should be light during medication, fasting cold, spicy, warm and dry fried sweet food, fasting hair such as seafood, trotters, pigeons, etc
(2) Tobacco and alcohol, stay up late;
(3) Maintain emotional stability, avoid sorrow and anger, avoid excessive tension;
(4) During the medication should avoid wind and cold, appropriate activities, and avoid strenuous exercise.

#### 2.3.2 Intervention group

We will give acupuncture treatment.

(1) Acupoints: Guan Yuan, Uterus, Sanyinjiao, Qihai, Taichong, Tianshu, Zhongji.
(2) Acupoint positioning: The acupoint positioning will refer to the 2006 National Standard of the People's Republic of China (GB/T12346-2006) “Name and Location of Acupoints”.
(3) Acupuncture needles: Huaying brand acupuncture needles (0.30 mm × 40 m) manufactured by Suzhou Medical Supplies Factory.
(4) Operation method: The patient is in a supine position, and the acupuncture site is wiped and disinfected with a 75% alcohol cotton ball. All the acupuncture points are 0.30 mm × 40 mm acupuncture needles, and 13 to 40 mm is directly pierced. We will refer to the operation of acupuncture in the textbook of “Acupuncture and Moxibustion Law” edited by Lu Shoukang. The local acid swelled and became angry, and evenly inserted and twisted three times in a small amount, leaving the needle for 30 minutes, and performing the needle once every 10 minutes during the needle retention, a total of three times. When removing the needle, the left thumb and forefinger hold the disinfected cotton ball lightly on the acupuncture site, and the right hand slowly lifts the needle until the needle is taken out subcutaneously. After the needle is released, press the needle hole to prevent bleeding.
(5) Treatment course: Leave the needle for 30 minutes each time, and treat three times a week, once on Monday, Wednesday, and Friday, for 24 times in 2 months.

### 2.4 2.4.Outcome measures

#### 2.4.1 Primary outcome measures

(1) The symptom severity of quality uterine fibroid (SS-QOL): The SS-QOL scale involves eight issues: increased menstrual flow, menstrual blood clots, prolonged menstrual periods, and irregular menstrual cycles Regularity, lower abdominal discomfort, frequent urination during the day, frequent urination at night and fatigue. Each question contains five options (1 = none, 2 = one point, 3 = mild, 4 = moderate, 5 = severe, 1–5 points, respectively, total score 8–40 points). SS-QOL scale total score = (actual total score − lowest possible total score)/possible total partition between  ×  100 (*Note*: the highest possible total score = 40, the lowest possible total score = 8, the possible total intervals = highest possible total score − lowest possible total score = 32). The higher the total score of the SS-QOL scale, the more severe the symptoms of the patient.
(2) The health-related quality of life of uterine fibroid (HR-QOL) questionnaire for UF: the HR-QOL scale involves six areas: disease concerns, activities, energy and mood, Life out of control, self-care, and sexual function. Includes 29 questions, each of which contains five options (exactly, often, sometimes, rarely, never). Total score of HR-QOL scale = (highest possible total score − actual total score)/total possible inter-zone  ×  1009. (*Note*: the highest possible total score = 145, the lowest possible total score = 29, the total possible partitions = the highest possible total score − the lowest possible total score = 16); the higher the total score of the HR-QOL scale, the higher the patient's quality of life.

#### 2.4.2 Secondary outcome measures

(1) Menorrhagia Questionnaire (MQ): The MQ scale is based on menstrual duration, menstrual volume, menstrual abdominal pain, and the impact of symptoms on daily activities. The higher the score, the more severe the symptoms.
(2) Overall Recovery Self-Scoring (OTE): The OTE scale is a seven-level assessment scale 1. Consider the characteristics of uterine fibroid patients and Chinese language habits of Chinese patients. This trial will use a seven-level evaluation of OTE to classify the symptoms of patients with uterine fibroids as either improving, or staying the same, or worsening than the last time. If symptoms improve, they will self-evaluate and score from a corresponding seven-point scale (no improvement, slight improvement, some improvement, half improvement, significant improvement, almost complete recovery, and complete recovery). If symptoms worsen, they will self-evaluate and score from a corresponding seven-point scale (no worsening, a little worsening, some worsening, half worsening, obvious worsening, almost complete worsening, and complete worsening).

### 2.5 Randomization and blinding

(1) Random numbers are generated by computer randomization for randomized grouping. The specific operation method is as follows: Number from small to large according to the order of the subjects’ diagnosis (the numbers used for numbering are two digits, and those with less than two digits are supplemented with 0).
(2) Use SPSS24.0 statistical software to randomly generate 60 random numbers; rank the 60 random numbers on a case-by-case basis, and finally divide these 60 random numbers into 1 group (intervention group) and 30 groups (control group) 30 cases; (see Appendix I for grouping results).
(3) Acupuncture treatment will be required in group 1 and Chinese patent medicine treatment will be used in two groups.
(4) Documents of random distribution plan will be kept. Each subject's treatment plan will be generated by a computer-generated random allocation sequence. According to the random data generated by the computer, the research supervisor put the paper grouped into 1 group into the envelope numbered 01, put the paper grouped into 2 into the envelope numbered 02, and put the paper into grouped number 03. Place envelopes of group 1 in the envelope ... After the envelopes are packed, return the sealed 60 envelopes to the research staff. When the first patient appears, determine whether to include the study according to the criteria and explain the relevant situation of the study to the patient. After obtaining the patient's consent, fill in the informed consent form and number the patients in the order of the patient's visit. The patient opens the corresponding numbered envelope and treats it according to the group treatment plan in the envelope.

### 2.6 Statistical analysis

SPSS for windows 24.0 statistical analysis software will be used for calculation, and normality test and homogeneity test of variance will be performed on each group of data. For measurement data in which the data conforms to the normal distribution, we will use the mean ± standard deviation. For non-normally distributed measurement data, the median ± quartile interval is used. General data comparison between the two groups using independent sample *t* test. Comparisons before and after treatment will be performed using *t* test for paired data. One-way analysis of variance will be used for comparison between groups. *X*^2^ test will be used for count data, and nonparametric rank sum test will be used for rank data. All statistical tests are two-sided. *P* < .05 indicates a significant difference.

### 2.7 Data management

Information obtained from the evaluation of each participant will be recorded on a paper print-out. The information will then be handwritten on a paper document case report form and entered into an Excel file for future statistical analyses. In accordance with the Personal Information Protection Act, the names of all participants will not be disclosed, and a unique identifier number given during the trial will be used to identify participants. All of the participants will be informed that the clinical data obtained in the trial will be stored in a computer and will be handled with confidentiality. The participants’ written consent will be stored by the principal investigator.

### 2.8 Ethics

This study will be approved by the Ethics Committee of Fuzhou hospital of traditional Chinese medicine. The study will be conducted under the Declaration of Helsinki principles, as well as following the norms of good clinical practice. Recruitment of patients has not started in this study. The study plan will be submitted to the ethics committee of the Fuzhou Hospital of Traditional Chinese Medicine. We will not start recruiting participants without the consent of the ethics committee. The protocol of this study has been registered in the Chinese Clinical Trial Registry with the number ChiCTR2000030438.

## 3 Discussion

UF is one of the most common diseases in gynecology, and the specific cause is not clear. Some scholars hold that estrogen or UF can increase rapidly during pregnancy.^[11]^ However, after treatment with gonadotrophins or a decrease in postmenopausal 2 concentration, UF can gradually shrink and become smaller. It can be inferred that estrogen can promote the occurrence and development of UF, and progestin antagonists can reduce the effect of UF.^[12,13]^ Therefore, we consider UF to be benign ovarian tumors. Modern medicine's treatment of this disease is based on the dependence of UF on sex hormones. Treatment with antiprogestin and estrogen drugs can reduce the volume of fibroids or slow the rate of increase in volume, thereby achieving the goal of alleviating clinical symptoms.^[14]^ Mifepristone is a commonly used treatment drug, but these drugs have corresponding disadvantages, that is, there is a risk that fibroids may increase in volume after discontinuation. And there is the possibility of increasing endometrial hyperplasia.^[15,16]^ TCM theory believes that acupuncture can stimulate the body and activate its own regulatory functions, thereby improving and correcting organ disorders and dysfunction.^[17]^ Acupuncture treatment can clear the meridian and regulate the meridian to restore the normal function of the viscera and meridian.^[18]^ Acupuncture at acupoints such as *Zhongji*, *Uterus*, *Guanyuan*, *Qihai*, *Sanyinjiao*, and *Taichong* can promote dredging the meridians, regulate the functions of the viscera, and return the functions of the viscera to normal. Modern research suggests that human life is dominated by nerves.^[19]^ One study suggested that acupuncture can release neurochemicals, usually enkephalin, dynorphin, or serotonin. Acupuncture may stimulate the gene expression of neuropeptides, restore normal nerve functions, and restore the muscle, blood vessels, viscera, and hormone secretion functions normal to the nerves.^[20,21]^ Acupuncture of connective tissue can transmit mechanical signals to connective tissue and adjust the metabolism of connective tissue, which may be the key to acupuncture treatment of UF. Studies have suggested that the growth of UF is regulated by a complex feedback loop between estrogen, progesterone, and growth factors.^[22]^ Acupuncture is used to regulate pelvic nerves and female sex hormone levels, improve blood circulation in the pelvic cavity, and enable UF to be effectively treated.^[23]^ Therefore, acupuncture as a treatment for UF is well-founded and may have long-term effects.

In order to meet the needs of the majority of women of childbearing age and to maintain fertility, acupuncture treatment of UF has a broad prospect for development. Summarized research is urgently needed for standardized complementary and alternative therapies. This trial intends to adopt a randomized, patient-blind, and blinded efficacy evaluator and data processor design scheme to compare the difference in the efficacy of acupuncture and fake needles to improve the symptoms of UF, and provide new evidence for acupuncture treatment of UF. It is hoped that in the future, we can effectively solve the pain and psychological burden of patients through the combined treatment of various medical schemes.

### 3.1 Trial status

At the time of manuscript submission, recruitment for the study is not yet started.

## Acknowledgments

The authors would like to thank all the trial participants. The authors are grateful for the support for this study: trial coordinating team, surgical staff, nurses, and research departments.

## Author contributions

MQZ, CW, and WH designed the study protocol and drafted the manuscript. CQS reviewed the study protocol and drafted the manuscript. YT is responsible for the statistical design and analysis as trial statistician. All authors carefully read and approved the final version of the manuscript. ZWP participated in the design and coordination of the study. All authors read and approved the final manuscript.

Cai Weng orcid: 0000-0001-8174-9280.

## Supplementary Material

Supplemental Digital Content
